# Novel mutations in the QRDR region *gyr*A gene in multidrug-resistance *Corynebacterium* spp. isolates from intravenous sites

**DOI:** 10.1007/s10482-019-01353-w

**Published:** 2019-11-18

**Authors:** Juliana Nunes Ramos, Talita Bernardo Valadão, Paulo Victor Pereira Baio, Ana Luiza Mattos-Guaraldi, Verônica Viana Vieira

**Affiliations:** 1 Instituto Nacional de Controle de Qualidade em Saúde (INCQS), Fundação Oswaldo Cruz (FIOCRUZ), Rio de Janeiro, Brazil; 2 Laboratório de Difteria e Corinebactérias de Importância Clínica (LDCIC), Faculdade de Ciências Médicas, Universidade do Estado do Rio de Janeiro, Rio de Janeiro, Brazil; 3 Laboratório Interdisciplinar de Pesquisas Médicas (LIPMED), Instituto Oswaldo Cruz, Fundação Oswaldo Cruz (FIOCRUZ), Av. Brasil, 4365, Pavilhão Cardoso Fontes, 10 andar, sala 17, Manguinhos, Rio de Janeiro, RJ CEP 21040-900 Brazil; 4 Laboratório Químico-Farmacêutico do Exército (LQFEx), Ministério da Defesa, Rio de Janeiro, Brazil

**Keywords:** *Corynebacterium*, Multidrug-resistance, Fluoroquinolones, *gyr*A mutation, QRDR

## Abstract

The resistance to fluoroquinolones in corynebacteria is due to mutations occurring in the quinolone-resistance-determining region (QRDR) of the *gyr*A gene encoding the enzyme gyrase A subunit. In recent years we can observe an increasing number of infections caused by multidrug-resistant *Corynebacterium striatum, Corynebacterium jeikeium* and *Corynebacterium urealyticum*, including wide range of disorders, such as invasive infections. In this study 14 *Corynebacterium* spp. isolated from intravenous sites were sequenced and new combinations of mutations in the QRDR of the *gyr*A gene were found in *C. jeikeium* and *C. urealyticum.* Nowadays, no study comparing mutations in this region and the susceptibility to fluoroquinolones in *C. jeikeium* and *C. urealyticum* has been described. All the isolates that showed double mutation (position 87 and 91) in the QRDR *gyr*A gene had high MIC to the fluoroquinolones tested.

The antibiotic resistance among *Corynebacterium* species has increased, sometimes leading to the use of vancomycin as the drug of choice (Yoo et al. 2015). Some *Corynebacterium* species belonging to human flora skin such as *Corynebacterium striatum, Corynebacterium jeikeium* and *Corynebacterium urealyticum* have expressed multidrug-resistance can cause a wide range of disorders as bacteremia, endocarditis, septicemia and others invasive infections mainly in immunocompromised patients (Bernard 2012).

Resistance to fluoroquinolones has become common in some bacterial pathogens. Analysis of the sequences of the QRDR of the *gyr*A gene in isolates of *C. striatum, Corynebacterium amycolatum,* and *Corynebacterium macginleyi* have shown that resistance to fluoroquinolones is associated with mutations of a spontaneous nature in this gene and depends on the number of mutations and the type of aminoacid that has been substituted (Sierra et al. 2005; Eguchi et al. 2008; Alibi et al. 2017).

In this study, we investigated 14 multidrug-resistant isolates of *C. striatum* (n = 7), *C. jeikeium* (n = 4) and *C. urealyticum* (n = 3) from blood (n = 10) and catheter segments (n = 4), during the period of 48 months (Aug. 2009 – Aug. 2013) of 13 hospitalised patients attended in two hospitals located at the metropolitan area of Rio de Janeiro, RJ, Brazil. All isolates were deposited in the bacteria collection: Coleção de Bactérias do Ambiente e Saúde of Fundação Oswaldo Cruz (CBAS/FIOCRUZ—www.cbas.fiocruz.br).

The identity of isolates was confirmed by 16S rRNA and *rpo*B genes sequencing (Baio et al. 2013). The antimicrobial susceptibility test by disk-diffusion method according to Brazilian Committee on Antimicrobial Susceptibility Testing (BrCAST) document showed that all isolates of *C. jeikeium*, *C. urealyticum* and three isolates of *C. striatum* were resistant to penicillin, gentamicin, clindamycin, erythromycin, rifampin and imipenem. Five isolates of *C. striatum* showed variable susceptible to gentamicin, rifampin and imipenem, but all isolates of corynebacteria were susceptible to linezolid, tetracycline and vancomycin. The minimum inhibitory concentration (MIC) using E-test strips (AB Biodisk, Sweden) for ciprofloxacin, levofloxacin and moxifloxacin was performed according to BrCAST document. Due to absence of levofloxacin breakpoints in this guideline, the interpretation of values was interpreted in accordance to criteria defined by BrCAST for *Staphylococcus* spp. (Alibi et al. 2017).

The sequences of the QRDR of the *gyr*A gene were compared to that of the quinolone-susceptible type strains *C. striatum* ATCC 6940, *C. jeikeium* ATCC 43734 and *C. urealyticum* DSM 7109 (Table 1). All type strains have codons for Ser-87 and Asp-91 in the QRDR region of the *gyr*A gene. The MIC for fluoroquinolones were compared to the mutations in QRDR of the *gyr*A gene. All *C. striatum* isolates showed mutations in the codon 87, only two isolates showed double mutation in codons 87 and 91. Two *C. jeikeium* isolates showed MIC > 32 µg/mL for all fluoroquinolones tested probably due to novel mutations in the QRDR *gyr*A gene, where in the codon for Ser-87 changed to Ile-87 and the codon Asp-91 to Tyr-91. The three *C. urealyticum* isolates showed double mutation in codons 87 and 91. Two isolates showed mutation of Ser-87 to Tyr-87 and one isolate of Ser-87 toVal-87. The codon Asp-91 was changed to Ala-91 (two isolates) and Tyr-91 (one isolate). All *C. urealyticum* isolates showed MIC > 32 µg/mL to ciprofloxacin, levofloxacin and moxifloxacin. All isolates *Corynebacterium* spp. with mutations in the codons 87 and 91 had the highest MIC for moxifloxacin.

**Table 1:** Relationship between mutations in the QRDR of the *gyr*A gene and the MIC for *Corynebacterium* spp

Species	Isolates/CBAS no	*Gyr*A (amino acids)		MIC (µg/mL)			Genbank no		
		Position 87	Position 91	CIP	LVX	MXF	*gyr*A	16S rRNA	*rpo*B
*C. striatum*	2130/CBAS 612	Tyr	Asp	2	1.5	0.75	MG010352	KJ855309	KR010642
	2296/CBAS 615	Phe	Ala	> 32	> 32	> 32	MG010359	KJ855313	KR010636
	2425/CBAS 620	Phe	Ala	> 32	> 32	> 32	MG010366	KM001911	KR010631
	2023/CBAS 618	Val	Asp	> 32	> 32	8	MG010347	JF342699	JF342707
	2230/CBAS 617	Val	Asp	> 32	> 32	8	MG010354	KJ855311	KR010641
	2237/CBAS 616	Val	Asp	> 32	> 32	4-6	MG010355	KJ855312	KR010640
	2308/CBAS 614	Val	Asp	> 32	> 32	6	MG010360	KJ934785	KR010635
	ATCC 6940	Ser	Asp	0.094	0.19	0.125	ACGE01000134	ACGE01000134	ACGE01000134
*C. jeikeium*	2325/CBAS 677	Ile	Asp	> 32	> 32	4–6	MH513932	MH510232	MH513925
	2509/CBAS 681	Ile	Asp	> 32	> 32	4	MH513935	MH510235	MH513928
	2443B/CBAS 679	Ile	Tyr	> 32	> 32	> 32	MH513933	MH510233	MH513926
	2444/CBAS 680	Ile	Tyr	> 32	> 32	> 32	MH513934	MH510234	MH513927
	ATCC 43734	Ser	Asp	0.125	0.19	0.125	ACYW01000075	ACYW01000075	ACYW01000075
*C. urealyticum*	2260/CBAS 675	Tyr	Ala	> 32	> 32	> 32	MH513936	MH510236	MH513929
	2287B/CBAS 676	Tyr	Ala	> 32	> 32	> 32	MH513937	MH510237	MH513930
	2431/CBAS 678	Val	Tyr	> 32	> 32	> 32	MH513938	MH510238	MH513931
	ATCC 43042	Ser	Asp	0.125	0.125	0.125	NC010545	NC010545	NC010545

*MIC* minimum concentration inhibitory, *CIP* ciprofloxacin, *LVX* levofloxacin, *MXF* moxifloxacin

Fluoroquinolones have been extensively used in the empirical treatment of urinary tract infections, including in Brazil (Hisano et al. 2015). These drugs accumulate in the organs of the body leading to the selection of spontaneous mutants in large populations that colonize the skin and mucous membranes, such as corynebacteria, which can cause nosocomial bacteremia (Sierra et al. 2005; Alibi et al. 2017). Studies have shown that increasing fluoroquinolones resistance rates in almost all bacterial species have limited empirical antimicrobial treatment options (Dalhoff 2012).

To our knowledge, no study comparing the mutations in the *gyr*A gene and the susceptibility to fluoroquinolones in *C. urealyticum* and *C. jeikeium* has been described. In summary, we report here the emergence of fluoroquinolone resistance in *Corynebacterium* species isolated from blood and catheter segments with novel mutations at amino acid positions 87 and 91 in QRDR *gyr*A gene producing high levels of resistance to ciprofloxacin, levofloxacin and moxifloxacin.
